# Antioxidant and Anti-atherogenic Properties of *Prosopis strombulifera* and *Tessaria absinthioides* Aqueous Extracts: Modulation of NADPH Oxidase-Derived Reactive Oxygen Species

**DOI:** 10.3389/fphys.2021.662833

**Published:** 2021-07-16

**Authors:** Isabel Quesada, Matilde de Paola, María Soledad Alvarez, María Belén Hapon, Carlos Gamarra-Luques, Claudia Castro

**Affiliations:** ^1^Consejo Nacional de Investigaciones Científicas y Técnicas (CONICET), Instituto de Medicina y Biología Experimental de Cuyo (IMBECU), Mendoza, Argentina; ^2^Facultad de Ciencias Médicas, Instituto de Bioquímica y Biotecnología, Universidad Nacional de Cuyo, Mendoza, Argentina; ^3^Facultad de Ciencias Exactas y Naturales, Universidad Nacional de Cuyo, Mendoza, Argentina; ^4^Facultad de Ciencias Médicas, Instituto de Fisiología, Universidad Nacional de Cuyo, Mendoza, Argentina

**Keywords:** atherosclerosis, oxidative stress, natural antioxidant, *Prosopis strombulifera*, *Tessaria absinthioides*

## Abstract

Despite popular usage of medicinal plants, their effects as cardiovascular protective agents have not been totally elucidated. We hypothesized that treatment with aqueous extract from *Prosopis strombulifera* (AEPs) and *Tessaria absinthioides* (AETa), Argentinian native plants, produces antioxidant effects on vascular smooth muscle cells (VSMCs) and attenuates atherogenesis on apolipoprotein E-knockout (ApoE-KO) mice. In VSMCs, both extracts (5–40 μg/ml) inhibited 10% fetal calf serum-induced cell proliferation, arrested cell in G2/M phase, reduced angiotensin II-induced reactive oxygen species (ROS) generation, and decreased NADPH oxidase subunit expression. In ApoE-KO mice, extracts significantly reduced triglycerides and lipid peroxidation [plasma thiobarbituric acid reactive substances (TBARS)], increased plasma total antioxidant status (TAS), and improved glutathione peroxidase activity in the liver. Under high-fat diet (HFD), both extracts were able to inhibit O_2_^–^ generation in the aortic tissue and caused a significant regression of atheroma plaques (21.4 ± 1.6% HFD group vs. 10.2 ± 1.2%^∗^ AEPs group and 14.3 ± 1.0%^∗^ AETa group; ^∗^*p* < 0.01). Consumption of AEPs and AETa produces antioxidant/antimitogenic/anti-atherosclerotic effects, and their use may be beneficial as a complementary strategy regarding cardiovascular disease therapies.

## Graphical Abstract

Schematic representation of the effect of *P. strombulifera* or *T. absinthioide* decoctions on VSMC and ApoE-KO mice. The combination of antimitogenic effect and antioxidant capacity of the extracts is effective to increase antioxidant status and reduce atheroma development modulating NADPH oxidase expression.

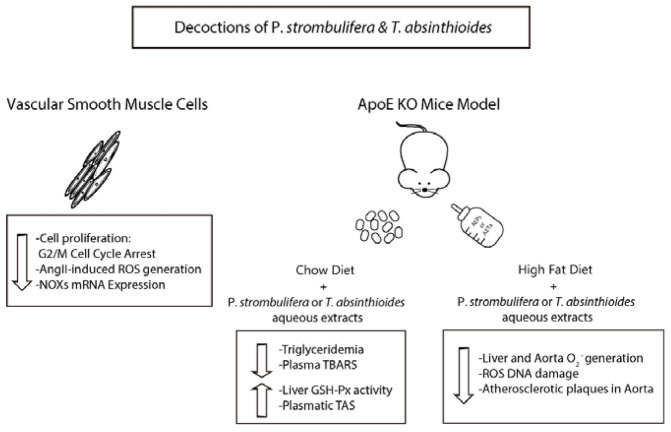

## Introduction

In recent years, the study of the beneficial effects on human health of the consumption of antioxidants contained in natural products has achieved great relevance (Chan and Chan, 2015; Siti et al., 2015; Griffiths et al., 2016; Jiang and Xiong, 2016). Given their wide structural and biological diversity, natural products represent the most abundant source of chemical structures, making them suitable as the basis for the rational design of drugs (Wenderski et al., 2015). A variety of plant materials are known to be natural sources of antioxidants, such as herbs, spices, seeds, fruits, and vegetables (Lourenco et al., 2019; Rodriguez-Garcia C. M. et al., 2019). Recent studies have been carried out on the potential properties of the native flora, finding plants whose aqueous and/or ethanol extracts have health effects and therapeutic properties against various chronic diseases (Ayeleso et al., 2017; Houghton, 2019; Rodriguez-Garcia C. et al., 2019).

Oxygen free radicals cause oxidative damage, which has been implicated in the initiation or progression of several chronic diseases, ranging from cancer (Zhou et al., 2019) to cardiovascular diseases (CVDs) (Siti et al., 2015) including atherosclerosis (Kattoor et al., 2017; Lankin and Tikhaze, 2017), hypertension (Sinha and Dabla, 2015), stroke (Rodrigo et al., 2013), heart failure (Lubrano and Balzan, 2020), and diabetes-associated heart dysfunction (Knapp et al., 2019). Indeed, reactive oxygen species (ROS) seems to be the common mechanism for the development of endothelial dysfunction, vascular inflammation, and arterial stiffness (Neves et al., 2018). Oxidative stress generally refers to state in which balance in the oxidant/antioxidant mechanisms is disrupted; therefore, an antioxidant therapy may be a valid approach to neutralize or scavenge free radical excess in a way to reestablish a proper and healthy cell metabolism (Wojcik et al., 2010; Pizzino et al., 2017).

*Prosopis* spp. and *Tessaria* spp., for example, are native plants capable of growing in arid and semiarid environments, which have long been used in traditional medicine (Chaudhary et al., 2018; Solanki et al., 2018; Utage et al., 2018; Cattaneo et al., 2019). Both species have been recognized for their antioxidant (Torres-Carro et al., 2019; Vasile et al., 2019), anti-inflammatory (Henciya et al., 2017; Yadav et al., 2018), and antitumoral properties (Hapon et al., 2014; Persia et al., 2017; Khodaei et al., 2018). Particularly, two species originally from Mendoza (Argentina), *Prosopis strombulifera* and *Tessaria absinthioides*, have an extensive and popular usage; however, their effects on CVDs remain unknown. The chemical characterization of the aqueous extract of *P. strombulifera* (AEPs) by ultrahigh-performance liquid chromatography–Q-Orbitrap–heated electrospray ionization–tandem MS (UHPLC–Q-OT–HESI–MS/MS) recently published identified 26 compounds, including five simple organic acids, four phenolic acids, four procyanidins, 11 flavonoids, and two oxylipins (Persia et al., 2020). On the other hand, aqueous extract of *T. absinthioides* (AETa) is characterized by a high content of phenolic acids and possesses many eudesmane-type sesquiterpenoids (Gomez et al., 2019). In addition, acute, subacute, and chronic oral administration toxicity for both aqueous extracts were discarded in accordance to the Organization for Economic Co-operation and Development (OECD) guidance (Hapon et al., 2014; Persia et al., 2017).

The present study was designed to elucidate the antimitogenic and antioxidant properties of AEPs and AETa on vascular smooth muscle cells (VSMCs). Whether these extracts have protective effects on atherosclerosis progression in apolipoprotein E-knockout (ApoE-KO) mice fed with high-fat diet (HFD) was also investigated.

## Materials and Methods

### Plant Material and Preparation of Soluble Fraction

*P. strombulifera* and *T. absinthioides* were collected in Lavalle County, Mendoza, Argentina (33°44′10′′S, 68°21′30.5′′W), and the voucher specimens were deposited in the Mendoza Ruiz Leal Herbarium as MERL-61824 (Ps) and MERL-61823 (Ta). Decoctions of *P. strombulifera* and *T. absinthioides* were prepared at 10% weight/volume, from 100 g of dried and milled plant (leaves), in 1 L of purified water. After 30 min of boiling, the decoctions were filtered and cooled for 24 h in a freezer at −20°C, and then three representative samples of 100 ml of each decoction were subsequently lyophilized in LA-B3 RIFICOR equipment (Buenos Aires, Argentina). The samples were stored in a freezer at −20°C until its use in the assays included in this work, in phenolic and flavonoid quantification, and in UHPLC–PDA–OT–MS analysis. Detailed phytochemical analyses were published by Persia et al. (2020) and Gomez et al. (2019).

### Isolation of Vascular Smooth Muscle Cells

VSMCs were isolated from aortas of 12-week-old Wistar rats according to a previously described technique (Castro et al., 2010). More details in Supplementary Material.

### Cell Viability and Proliferation

Plated in 24-well plates were 1 × 10^4^ VSMCs and serum-starved for 24 h. Then, cells were treated with 2.5, 5, 10, 20, or 40 (μg/ml) of AEPS or AETA in either 0.1 or 10% fetal calf serum (FCS) DMEM/F12 during 24–48 h. Cell behavior was measured using a CellTiter Non-radioactive cell proliferation Assay Kit (G4001; Promega, Madison, WI, United States). More details in Supplementary Material.

### Flow Cytometry

Serum-starved VSMCs were stimulated with growth media (10% FCS) alone or co-incubated with AEPs or AETa (5 and 20 μg/ml) during 24 h. Then, cells were harvested and fixed with ice-cold 70% ethanol overnight. The fixed cells were centrifuged and treated with 0.1 mg/ml of RNase A and 50 μg/ml of propidium iodide for 30 min at room temperature. To determine the cell population distribution of fluorescence intensity, a FACS Aria II flow cytometer (Becton Dickinson, Franklin Lakes, New Jersey, United States) was used. For each sample, at least 10,000 nuclei were analyzed. DNA histograms were analyzed by FCS express 7.06.0015 *DeNovo* software. Two separate experiments were performed with similar results.

### Measurement of Reactive Oxygen Species in Intact Cells

Intracellular ROS levels were measured using the FluoroProbe CM-H_2_DCFDA (C400; Invitrogen, Carlsbad, CA, United States). VSMCs were cultured in a 24-well plate until confluence and then serum deprived for 24 h. AEPs and AETa (2.5, 5, 10, 20, or 40 μg/ml) were applied during 1 h. Basal fluorescence was measured, and then 100 nM of angiotensin II (AngII) (A9525; Sigma-Aldrich Corp., St. Louis, MO, United States) was added. Fluorescence of stimulated cells was measured continuously during 40 min on a microplate fluorometer (Fluoroskan Ascent, Labsystems, Helsinki, Finland), λ_ex_ = 485 nm and λ_em_ = 538 nm.

To determine intracellular O_2_^–^ production in VSMCs, dihydroethidium (DHE; Invitrogen) staining was performed. VSMCs were pre-incubated for 1 h with AEPs or AETa (5 or 20 μg/ml), stimulated with AngII (100 nM, 2 h), and incubated with DHE (5 μM) for 30 min at room temperature. Cells were imaged by fluorescence microscopy (Eclipse TE300 inverted microscopy, Nikon Instruments Inc., Melville, NY, United States), and the signal was quantized with ImageJ 1.50i software. To confirm that cell incubation with extracts affects ROS generation, control experiments with antioxidant agents including apocynin (a ROS scavenger; 100 μmol/L) and diphenylene iodonium chloride (DPI; a flavoprotein inhibitor) 10 μmol/L were carried out.

### Quantitative Reverse Transcription–Polymerase Chain Reaction Analysis

Total RNA was isolated with TRIzol (Invitrogen, 15596026) from VSMCs stimulated with AngII during 4 h and previously treated with or without AEPs or AETa (20 μg/ml). Real-time qPCR was performed with cDNA samples and EVA Green (31000; Biotium, Fremont, CA, United States) using a Rotor-Gene 6000 Series Software version 1.7 (Corbett Life Science, Sydney, NSW, Australia). The gene expression levels of NADPH oxidase (NOX) subunits NOX2 and NOX4 were normalized to 18S. The mRNA levels were expressed as a ratio, using the delta-delta method for comparing relative expression results between treatments. Relative expression was calculated as 2^–ΔΔCt^. More details in Supplementary Material.

### Western Blotting Analysis

VSMCs were pretreated with AEPs or AETa (20 μg/ml) and then stimulated during 20 h with AngII (100 nm). Total or fractionated proteins were obtained with a Subcellular Protein Fractionation Kit (#78840; Thermo Fisher Scientific, Waltham, MA, United States). Protein content was determined with Bradford protein assay reagent, and lysates (25 μg) were separated by 10% sodium dodecyl sulfate–polyacrylamide gel electrophoresis (SDS-PAGE), transferred to polyvinylidene difluoride (PVDF) membrane (Amersham, GE Healthcare, Buckinghamshire, United Kingdom), and probed with antibodies against NOX2 (1:500; #611414; BD Biosciences, San Jose, CA, United States) and NOX4 (1:500; Santa Cruz Biotechnology, Dallas, TX, United States). Detection was accomplished using horseradish peroxidase-coupled anti-rabbit or anti-mouse (1:2,000; The Jackson Laboratory, Bar Harbor, ME, United States) IgG antibody for 1 h at room temperature. Signal was detected using BioLumina (Kalium Tech, Buenos Aires, Argentina) detection system. Immunoblot signals were quantified by NIH ImageJ using β-actin expression as loading control.

### Animals and Study Design

**SCHEME 1:**
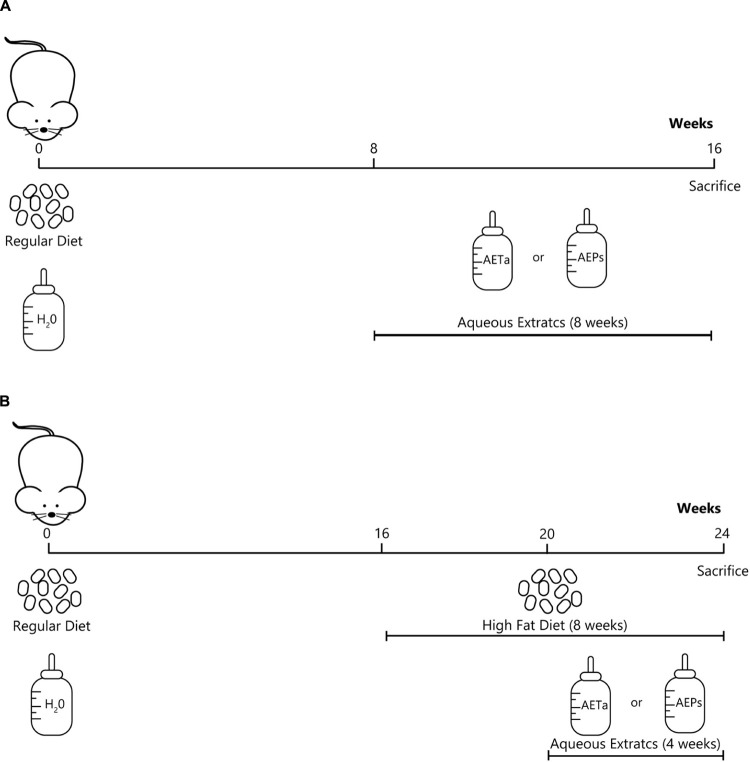
Experimental design showing the groups of animals, the type of diets and study-time points (weeks). **(A)** Scheme to evaluate the effects of *P. strombulifera* or *T. absinthioides* on biochemical parameters, and their role on preventing oxidative stress. **(B)** Scheme to assess the role of *P. strombulifera* or *T. absinthioides* on high-fat diet-induced atheroma development.

The experiments were carried out according to institutional guidelines for animal experiments, approved by the Institutional Committee for the Care and Use of Laboratory Animals (CICUAL) of Facultad de Ciencias Médicas, Universidad Nacional de Cuyo (Aval approval # 77/2016). The study design that was followed is shown in Scheme 1A. C57BL/6J ApoE-KO mice aged 2 months (The Jackson Laboratory, Bar Harbor, ME, United States) were grouped-housed, had unrestricted access to water and standard chow (GEPSA, Cordoba, Argentina), and maintained on a 12-h light/dark cycle. Mice were fed a normal diet and treated 8 weeks with AEPs or AETa (150 or 300 mg/kg/day, respectively) in the drinking water. During this time, body weight, diet, and water intake control were monitored. Blood samples were collected after 4-h fasting period by cardiac puncture under anesthesia (ketamine 80 mg/kg–midazolam 5 mg/kg). The glucose concentrations were measured immediately after blood collection with an enzymatic kit (1400101; Wiener Lab^®^, Rosario, Argentina). Plasma total cholesterol (TC) and triglycerides (TGs) were determined using colorimetric reactions with commercial kits (750220 and 791020, respectively; GT Lab, Buenos Aires, Argentina). As an indicator of lipid peroxidation, plasma malondialdehyde (MDA) was determined using the thiobarbituric acid reactive substances (TBARS) assay with own laboratory protocol. Total antioxidant status (TAS) in plasma was performed using a commercial kit (Randox Laboratories Ltd., Crumlin, United Kingdom) according to the manufacturer’s instructions. Trolox [(±)-6-hydroxy-2,5,7,8-tetramethylchromane-2-carboxylic acid] was used for calibration curve. Results are expressed as mmol equivalents of Trolox/L.

### Determination of Tissue Glutathione Peroxidase Activity and NADPH Oxidase-Derived Superoxide

For the antioxidant enzyme determination, liver was homogenized in a proportion of 200 mg/ml of homogenization buffer (phosphate buffer plus cOmplete Protease Inhibitor 1,000×, Roche Diagnostics, Basel, Switzerland) and centrifuged at 10,000 × g for 15 min at 4°C to eliminate cellular organelle debris. Supernatant was used for the determination of the antioxidant enzyme glutathione peroxidase (GPx) activity with a Randox Laboratories commercial kit. The protein concentration was determined using the Bio-Rad Protein Assay (Bio-Rad Laboratories, Hercules, CA, United States).

Tissue superoxide (O_2_^–^) production was calculated from the initial linear rate of superoxide dismutase (SOD)-inhibitable cytochrome *c* reduction assay quantified at 550 nm using the extinction coefficient of 21.1 mM^–1^ cm^–1^ as previously described (Molshanski-Mor et al., 2007). Superoxide production is expressed as nM O_2_^–^min/mg protein using the extinction coefficient. More details in Supplementary Material. As any electron donors can also reduce cytochrome *c*, the assay was performed in the presence of SOD, which removes superoxide. Only the SOD-inhibitable signal truly reflects superoxide production. Data are presented as nmoles O_2_^–^min/mg protein generated in the presence of SOD or DPI, a general flavoprotein inhibitor.

### Immunofluorescence Staining

Sections (5 μM) of 4% paraformaldehyde-fixed, paraffin-embedded aortic tissues were subjected to immunofluorescence staining. The sections were incubated with PBS plus 0.3% Triton X-100 (TPBS) for 5 min at room temperature, washed three times with PBS, blocked with 1% calf serum/TPBS for 1 h at room temperature, and incubated primary antibodies against 8-hydroxy-2′-deoxyguanosine (8-OHdG) conjugated to fluorescein isothiocyanate (FITC) (ab183393, 1:500; Abcam, Cambridge, United Kingdom) overnight at 4°C. The stained specimens were mounted and observed under fluorescence microscope (Olympus, Tokyo, Japan). Image analysis was performed by ImageJ 1.50i software.

### Atherosclerotic Lesion Measurement

The study design that was followed is shown in Scheme 1B. C57BL/6J ApoE-KO mice, 4 months old, were randomly divided into four groups (*n* = 6 each group), as follows: control diet (Control), HFD (standard chow supplemented with 30% bovine fat), HFD plus AEPs (150 mg/kg/day), and HFD plus AETa (300 mg/kg/day). HFD was administered during 8 weeks, and both extracts were administered in the drinking water during the last 4 weeks. All mice were sacrificed, and liver and whole aortas were excised. Each artery was fixed with 4% paraformaldehyde for 2 h and then placed in a 30% sucrose solution for 24 h. The anthropometric measurement of atherosclerotic lesions was determined according to Quesada et al. (2018).

### Statistical Analyses

Statistical analysis was performed using the GraphPad Prism 6.0 software. A *p*-value < 0.05 was considered statistically significant. Parametric or non-parametric tests were used according to variance homogeneity evaluated by F or Barlett’s tests. One-way ANOVA followed by Tukey’s multiple-comparison *post-test* was performed. Data represent the mean ± standard error of the mean (SEM) of three independent experiments for VSMC assays and three independent animal protocols.

## Results

### Anti-proliferative Effect of *Prosopis Strombulifera* and *Tessaria absinthioides*

**FIGURE 1:**
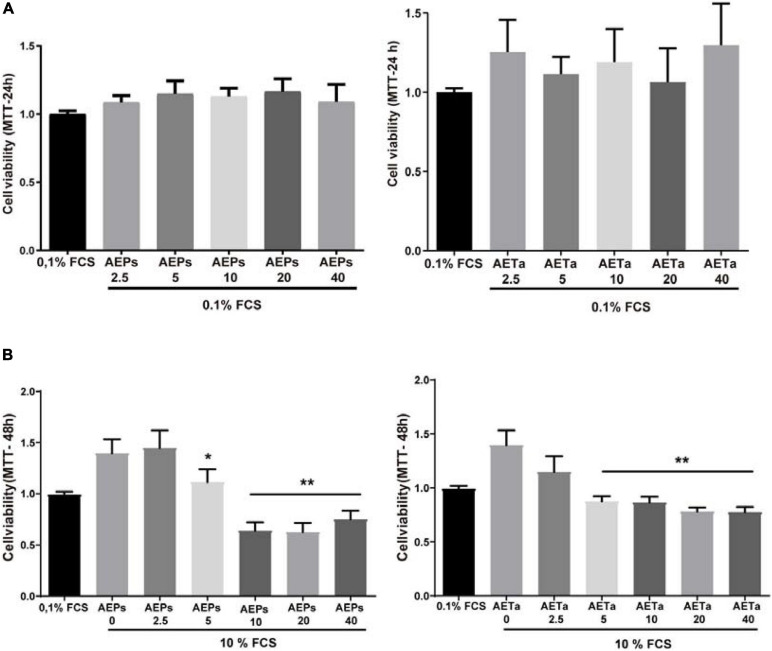
Effect of *Prosopis strombulifera* and *Tessaria absinthioides* on VSMC viability and proliferation. **(A)** VSMCs were incubated with 0.1% FCS containing 0, 2.5, 5, 10, 20, or 40 μg/ml AEPs or AETa during 24 h. The MTT assay revealed that none of the concentrations of any of the aqueous extracts used in the assay showed a cytotoxic effect. **(B)** Cells were treated with 0, 2.5, 5, 10, 20, or 40 μg/ml of AEPs or AETa and stimulated with 10% FCS. MTT assay revealed a dose-dependent reduction in cell proliferation after exposure to 5, 10, 20, or 40 μg/ml of AEPs or AETa. Data are shown as the mean ± SEM of three independent experiments. *p ≤ 0.01; **p ≤ 0.001 compared with the 10% FCS group. VSMC, vascular smooth muscle cell; FCS, fetal calf serum; AEPs, aqueous extract of *P. strombulifera*; AETa, aqueous extract of *T. absinthioides*.

**FIGURE 2:**
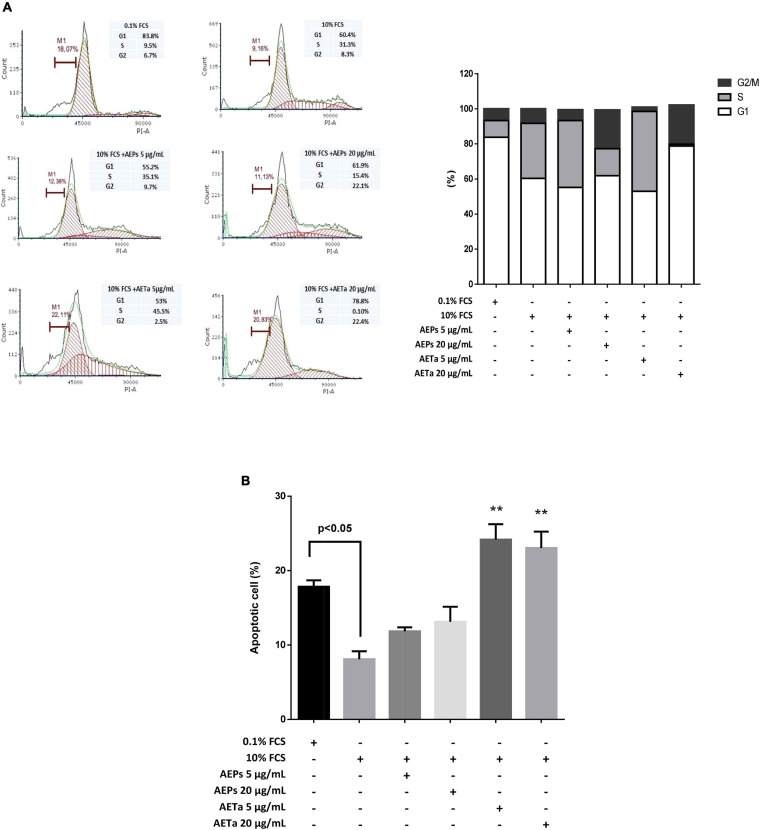
Effect of *Prosopis strombulifera* and *Tessaria absinthioides* on cell cycle progression. **(A)** DNA content staining with propidium iodide was analyzed by flow cytometry after 24-h incubation of treatment. Percentage of cell population in G1, S, and G2 phases of the cell cycle is shown. VSMC culture in 10% FCS and treated with AEPs and AETa (20 μg/ml) resulted in accumulation of cells at the G2 phase. **(B)** Apoptotic cell percentage was evaluated by sub-G0 population. AETa (5 and 20 μg/ml) treatments resulted in VSMC apoptosis induction after 24-h incubation. Data are shown as the mean ± SEM of two independent experiments. **p ≤ 0.01 compared with 10% FCS group. VSMC, vascular smooth muscle cell; FCS, fetal calf serum; AEPs, aqueous extract of *P. strombulifera*; AETa, aqueous extract of *T. absinthioides*.

Our first goal was to establish whether AEPs or AETa induced any harmful effect on VSMCs in culture. We tested a wide range of different concentrations of extracts (5–40 μg/ml) dissolved in 0.1% FCS. None of the concentrations tested affected cell viability when VSMCs were grown in medium containing a low concentration of FCS (0.1%, Figure 1A). Then, we stimulated VSMCs with 10% FCS during 48 h to promote cell proliferation, and we found that concentrations of 5–40 μg/ml of AEPs or AETa were able to affect VSMC proliferation (Figure 1B) assessed by MTT assay. In order to elucidate the molecular mechanisms of the antimitogenic effect of the extracts, we performed a cell cycle progression analysis by flow cytometry. VSMCs treated with 10% FCS showed an increase in the S phase population and a decrease in the G1 phase population as compared with 0.1% FCS control group (Figure 2A). A low concentration of either extract had no effect on 10% FCS-induced VSMC growth. After treatment with AEPs or AETa (20 μg/ml), the percentage of S phase cells was significantly reduced (15% for AEPs and 0.1% for AETa vs. 31.3% for 10% FCS), and the G2 phase percentage was increased (22.1% for AEPs and 22.4% for AETa vs. 8.3% for 10% FCS). These results indicate that AEPs or AETa (20 μg/ml) arrests VSMCs in the G2/M phase of the cell cycle. We also evaluated if the extracts were capable of inducing apoptosis, and we compared the percentages of cell on sub-G0 phase by flow cytometry. We found that AEPs produce a similar quantity of apoptotic cell as 10% FCS and less apoptosis that 0.1% FCS (Figure 2B). Remarkably, VSMCs growing in 10% FCS medium and treated 24 h with AETa (5 or 20 μg/ml) exhibited a greater number of cells in the sub-G0 phase, which would indicate that this extract could act as an inducer of apoptosis.

### Antioxidant Properties of *Prosopis Strombulifera* and *Tessaria absinthioides* in Vascular Smooth Muscle Cells

**FIGURE 3:**
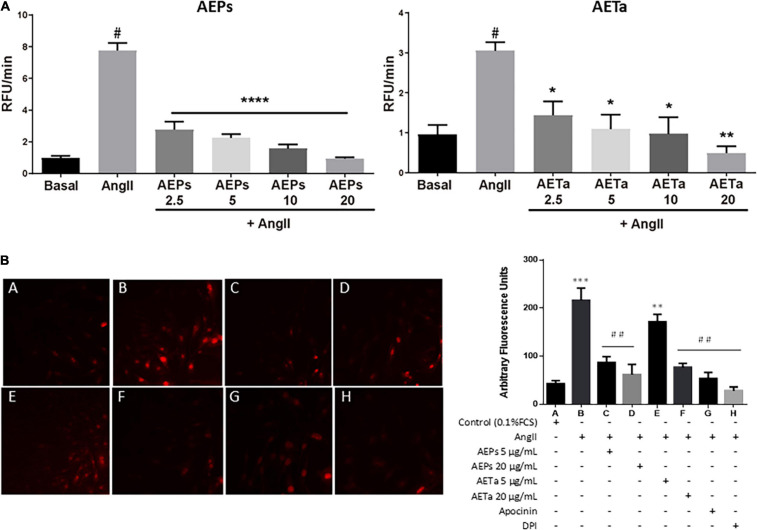
Effect of *Prosopis strombulifera* and *Tessaria absinthioides* on angiotensin II-induce ROS generation. **(A)** VSMC were pre-exposed to 0, 2.5, 5, 10, or 20 μg/ml of either AEPs or AETa, during 1 h prior to AngII (100 nM) addition. Results are presented as the AngII-induced changes in CM-H_2_DCFDA fluorescence after 40 min, calculated as the difference between the stimulated response and the basal value. **(B)** Intracellular superoxide anion generation: VSMC culture in 0.1% FCS (control A) or treated with AngII (100 nM) alone (B) or with AEPs 5 μg/ml (C) and 20 μg/ml (D); AETa 5 μg/ml (E) and 20 μg/ml (F); apocynin (G), or DPI (H); and stained with dihydroethidium (DHE). Red color fluorescence due to oxidant stress was observed using a fluorescent microscope as described in section “Materials and Methods.” Data are shown as the mean ± SEM of 3 independent experiments. *p ≤ 0.05, **p ≤ 0.01, ***p ≤ 0.001, ****p ≤ 0.0001 vs Control; ^#^*p* ≤ 0.01, ^##^*p* ≤ 0.001 vs Ang II. ROS, reactive oxygen species; VSMC, vascular smooth muscle cell; FCS, fetal calf serum; AEPs, aqueous extract of *P. strombulifera*; AETa, aqueous extract of *T. absinthioides*; DPI, diphenylene iodonium chloride.

To investigate the antioxidant action of AEPs and AETa, we measured ROS generation in AngII-stimulated VSMCs pretreated with the extracts using FluoroProbe CM-H_2_DCFDA (Figure 3A) or DHE (Figure 3B). The results showed that the addition of 2.5 up to 20 μg/ml of either AEPs or AETa significantly decreased intracellular ROS level induced by AngII. As seen in Figure 3B, DHE-derived fluorescent signals in VSMCs were increased by AngII compared with control (*p* < 0.0001). AEPs (5 and 20 μg/ml) and AETa 20 μg/ml significantly inhibited AngII-induced superoxide production (*p* < 0.001) in VSMCs, similar to apocynin and DPI effects. Our results suggest a strong antioxidant capacity for both aqueous extracts.

**FIGURE 4:**
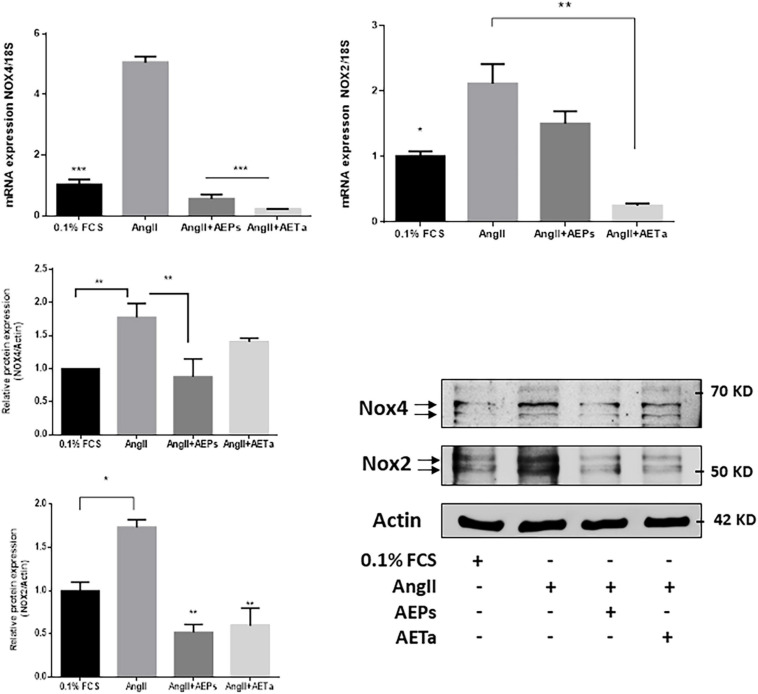
*Prosopis strombulifera* and *Tessaria absinthioides* downregulate NOXs expression. Relative mRNA expression was calculated as 2^–ΔΔCt^. Data are means ± SEM. *P ≤ 0.05, **P ≤ 0.01; ***P ≤ 0.001 vs AngII. AEPs (Aqueous extract of *Prosopis strombulifera*); AETa (Aqueous extract of *Tessaria absinthioides*). Nox2 and Nox4 protein expression was examined in VSMC pretreated with AEPs or AETa and stimulated with AngII. Graph represents quantification of all data (*n* = 3). *P ≤ 0.05; **P ≤ 0.01.

It is well established that the major and more specific enzymatic source of ROS in the vascular wall is NOX; and the isoforms NOX1, NOX2, and NOX4 are predominantly expressed in VSMCs (Garcia-Redondo et al., 2016). For this reason, we next evaluated the expression of NOX isoforms in VSMCs exposed to AngII, pretreated with AEPs or AETa. As is shown in Figure 4 (top panel), AngII-induced NOX4 expression is significantly downregulated by AEPs (both mRNA and protein expression), and AETa promoted decrease in NOX4 mRNA, which did not translate to protein expression. In addition, the expression of NOX2 enhanced by AngII was downregulated by AEPS (protein level) and significantly diminished by both AETa mRNA and protein levels (Figure 4, bottom panel).

### Effect of *Prosopis Strombulifera* and *Tessaria absinthioides* on Biochemical Parameters

**FIGURE 5:**
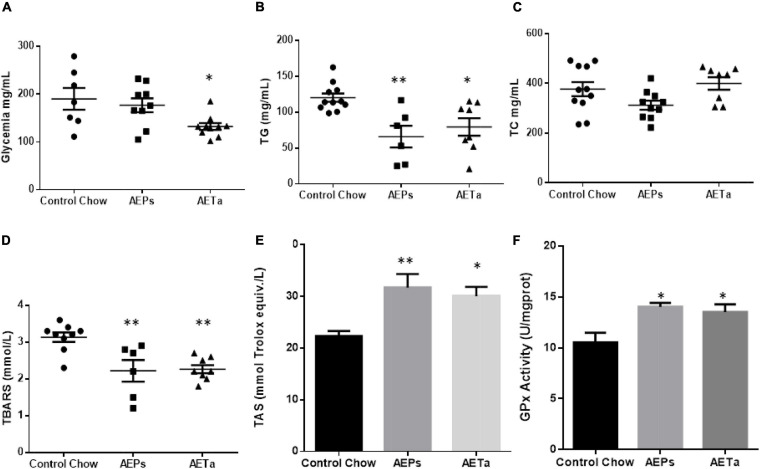
Effect of *Prosopis strombulifera* or *Tessaria absinthioides* on **(A)** fasting glycemia, **(B)** triglyceridemia, **(C)** total cholesterolemia, **(D)** plasma TBARS, **(E)** total antioxidant status (TAS) in plasma, and **(F)** liver GPx activity. ApoE-KO mice were fed with control chow, or AEPs or AETa in the drinking water (150 or 300 mg/kg/day, respectively) during 8 weeks. Data are means ± SEM (*n* = 6–9 mice per group); ^∗^*p* ≤ 0.05; ^∗∗^*p* ≤ 0.01 vs. control chow. TBARS, thiobarbituric acid reactive substances; ApoE-KO, apolipoprotein E-knockout; AEPs, aqueous extract of *P. strombulifera*; AETa, aqueous extract of *T. absinthioides*.

Using an experimental hypercholesterolemia animal model such as ApoE-KO mice, the effects of chronic intake of AEPs or AETa were investigated. The administration of AETa (300 mg/kg/day) during 8 weeks significantly reduced blood glucose levels as compared with the control chow group (190 ± 23 mg/dl (control group) vs. 132 ± 7 mg/dl (AETa group); *p* = 0.022 Figure 5A). No change was found with AEPs intake, in plasmatic glucose level. On the other hand, both extracts significantly reduced plasma triglyceride levels as compared with control chow mice [119 ± 7 mg/dl (control) vs. 47 ± 13^∗^ mg/dl (AEPs) and 50 ± 10^∗^ mg/dl AETa; ^∗^*p* < 0.0001, Figure 5B], while TC levels were not affected (Figure 5C).

### Role of *Prosopis strombulifera* and *Tessaria absinthioides* in Preventing Oxidative Stress

To address the capacity of the extracts on preventing systemic oxidative stress, we evaluated the level of lipid peroxidation and the TAS in plasma. We found a significant decrease in plasmatic TBARS in animals treated with AEPs or AETa [3.13 ± 0.13 mmol/L (control) vs. 2.21 ± 0.29 mmol/L (AEPs) and 2.26 ± 0.11 mmol/L (AETa); ^∗∗^*p* < 0.01] (Figure 5D). Simultaneously with reduced MDA levels, significantly higher levels of TAS were also found [22.3 ± 0.9 (control) vs. 31.7 ± 2.6 (AEPs) *p* < 0.01 and 30.1 ± 1.8 (AETa) *p* < 0.05 mmol of Trolox equiv./L] (Figure 5E). Interestingly, GPx activity, a measure to evaluate the level of antioxidant capacity in the liver, showed a significant increase in animals treated with the extracts [10.5 ± 0.9 U/mg (control) vs. 14.0 ± 0.9^∗^ U/mg (AEPs) and 13.5 ± 0.8^∗^ U/mg AETa; ^∗^*p* = 0.022; Figure 5F].

### Role of *Prosopis strombulifera* and *Tessaria absinthioides* on High-Fat Diet-Induced Atheroma Development

**FIGURE 6:**
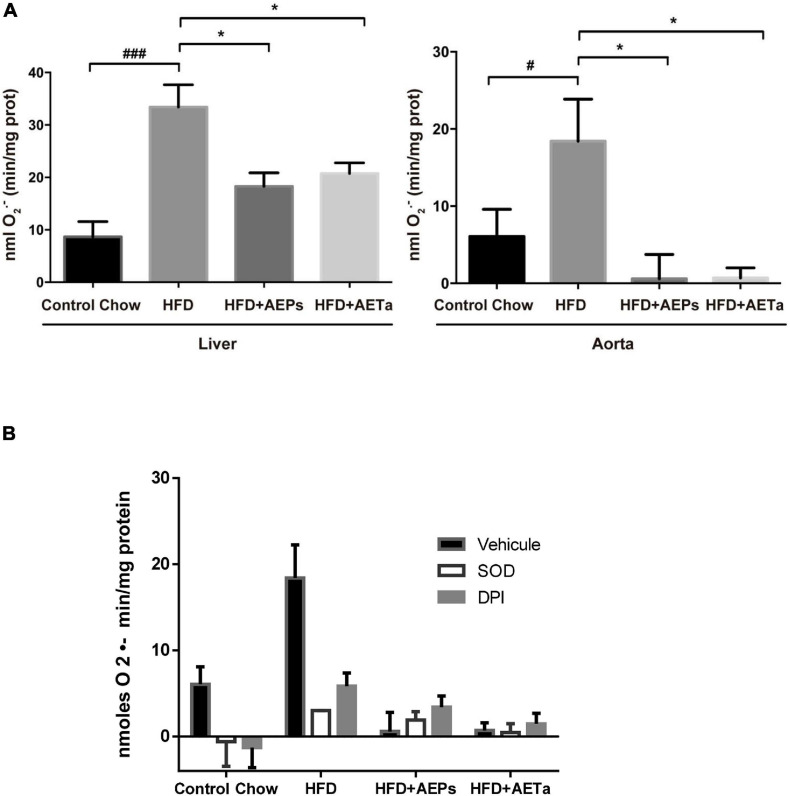
Effect of *Prosopis strombulifera* or *Tessaria absinthioides* on NOX-derived superoxide. **(A)** NOX-derived superoxide was measured by cytochrome *c* reduction assay in homogenates of liver and aortas from ApoE-KO mice fed a control diet or high-fat diet (HFD) alone or treated with AEPs or AETa (150 or 300 mg/kg/day, respectively) in the drinking water in the last 4 weeks. Data are means ± SEM (*n* = 4–6 mice per group). ^#^*p* ≤ 0.05 and ^###^*p* ≤ 0.001 vs. control chow; *p ≤ 0.05 vs. HFD. **(B)** Validation of superoxide dismutase (SOD)- and diphenylene iodonium chloride (DPI)-inhibitable superoxide production. As any electron donors can also reduce cytochrome *c*, the assay must therefore also be performed in the presence of SOD, which removes superoxide. Only the SOD-inhibitable signal truly reflects superoxide production. Here, we present the nmoles O_2_^–^min/mg protein generated by aorta tissue in the presence of SOD or DPI, a general flavoprotein inhibitor. AEPs, aqueous extract of *P. strombulifera*; AETa, aqueous extract of *T. absinthioides*; NOX, NADPH oxidase; ApoE-KO, apolipoprotein E-knockout; SOD, superoxide dismutase; DPI, diphenylene iodonium chloride.

**FIGURE 7:**
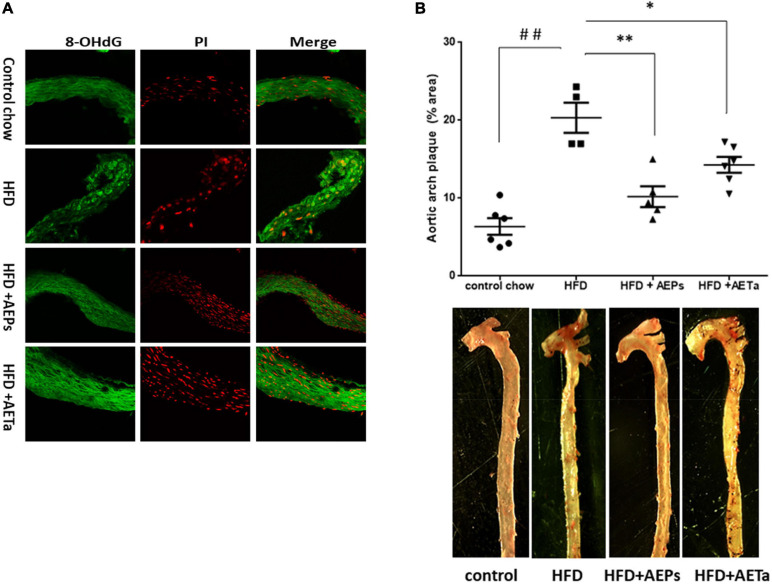
Effect of *Prosopis strombulifera* or *Tessaria absinthioides* on the progression of atherosclerosis. ApoE-KO mice were fed during 8 weeks with control diet (CD), high-fat diet (HFD), and HFD plus AEPs or AETa (150 or 300 mg/kg/day, respectively) in the drinking water in the last 4 weeks. After sacrifice, whole aortas were excised to determine **(A)** expression of 8OH-dG, a marker of oxidative damage of DNA. Representative photomicrographs of cross sections of the aorta of mice submitted to immunofluorescence reaction for 8-OHdG; PI, propidium iodide as nuclear marker. **(B)** Atheroma plaque area in the aortic arch and thoracic portion. Anthropometric measure was performed by staining with oil-red O. Plaque area in the images was quantified using the ImageJ 1.50i software. Data are means ± SEM (*n* = 4–6 mice per group). ^##^*p* < 0.001 vs. control chow; ^∗^*p* < 0.05 and ^∗∗^*p* < 0.001 vs. HFD. ApoE-KO, apolipoprotein E-knockout; AEPs, aqueous extract of *P. strombulifera*; AETa, aqueous extract of *T. absinthioides*.

An additional group of ApoE-KO mice were fed a HFD in order to exacerbate the pro-atherogenic state. Eight weeks of feeding HFD increased TC and triglycerides in ApoE-KO mice, compared with animals in control chow, and 4 weeks’ treatment with either extract was not enough to avoid the rise of these parameters (Table 1). However, 4 weeks’ treatment was enough to significantly reduce NOX-derived superoxide anion generation induced by HFD in the liver of ApoE-KO mice and almost completely removed superoxide anion generation in aortas (Figure 6A), demonstrating that both extracts have a potential ROS scavenging activity. As any electron donors can also reduce cytochrome *c*, the assay was performed in the presence of SOD, which removes superoxide. Only the SOD-inhibitable signal truly reflects superoxide production. Figure 6B shows nmoles O_2_^–^min/mg of protein generated by aorta tissues in the presence of SOD or DPI. As a marker of DNA oxidative damage induced by ROS, we detected immunofluorescence expression for 8-OHdG. Aortas from HFD-fed mice showed a stronger 8-OH-dG stain compared with aortas from mice fed control chow, and in the aortic wall of mice treated with HFD plus AEPs or AETa, 8-OH-dG stain was markedly weaker (Figure 7A). Next, we evaluated the atheroma development in our model. HFD showed an increase in atherosclerotic plaque size in aortas compared with control chow animals [6.37 ± 1.06% total area (control) vs. 20.33% ± 1.94% total area (HFD); ^∗∗^*p* < 0.001 Figure 7B], which was remarkably decreased in mice fed HFD plus AEPs (10.2 ± 1.33%^∗∗^ total area; ^∗∗^*p* < 0.01) or AETa (13.72 ± 1.02%^∗^ total area, ^∗^*p* < 0.05).

**TABLE 1:** Average plasma glucose and lipid levels in ApoE-KO mice fed control diet (CD), high-fat diet (HFD), and HFD with AEPs or AETa.

	CD	HFD	HFD + AEPs	HFD + AETa
Glycemia (mg/dl)	250 ± 15	311 ± 15*	364 ± 18*	309 ± 33*
Total cholesterol (mg/dl)	429 ± 25	624 ± 21**	701 ± 19**	595 ± 52**
Triglycerides (mg/dl)	128 ± 8	221 ± 26*	225 ± 18*	200 ± 17*

*Values are mean (n = 5–7) ± SEM. Animals were fed 8 weeks with HFD and treated with AEPs or AETa in the last 4 weeks.*

ApoE-KO, apolipoprotein E-knockout; AEPs, aqueous extract of Prosopis strombulifera; AETa, aqueous extract of Tessaria absinthioides.

*p = 0.05; **p = 0.01 vs. control diet (CD).

## Discussion

New strategies of prevention and treatments that reduce several risk factors of atherosclerosis and CVD are extremely necessary, and in this context, the use of native plants could be beneficial; however, this strategy deserves an in-depth analysis. Different properties of *P*. *strombulifera* and *T. absinthioides* and their health benefits (mainly antioxidant, antimicrobial, cytotoxic, and antitumoral effects) have been confirmed through *in vitro* and *in vivo* studies (Sharifi-Rad et al., 2019; Torres-Carro et al., 2019; Persia et al., 2020), but their beneficial effects on cardiovascular health have been less explored in *in vivo* studies. Here, we demonstrated that AEPs or AETa have an antimitogenic effect on VSMCs, as both extract reduce cell cycle progression by promoting G2 phase arrest and especially AETa acts as a potential inducer of apoptosis. To our knowledge, the current study reported for the first time a molecular mechanism of the effect of AETa on VSMC proliferation. The anti-proliferative properties of AEPs were studied and published in a previous work by Hapon et al. (2014). AEPs exhibited antiproliferative effect on tumoral cells (HCT-116 and MCF-7) by reduction of PCNA protein expression. When LC50 concentrations were used, AEPs induced necrosis [evidenced by the increase in extracellular lactate dehydrogenase (LDH) activity], apoptosis (increased cPARP protein expression), and clonogenic survival diminution. These results showed that *P. strombulifera* is a promising natural product for cancer research and treatment. On the other hand, AETa exhibits cytotoxicity against cancer cell line (Persia et al., 2017), but until now the molecular mechanisms involved in the antiproliferative capacity of AETa had not been reported.

We also demonstrated that both extracts prevent AngII-derived ROS production and modify the expression of NOX subunits. AEPs acts as a strong inhibitor of AngII-induced NOX4 expression (both mRNA and protein), while both extracts significantly inhibited NOX2 protein expression.

The excess of NOX-derived ROS is an important event in CVD, and AngII is a potent regulator of cardiovascular NOX system as an inducer of increase in NOX−mediated O${}_{2}^{•–}$ production in the cells (Chakraborti et al., 2019). Some natural phytochemical compounds, such as polyphenols, produce a direct scavenging of free radicals or have some potential inhibitory effect on NOX activity, as it is reviewed in Maraldi (2013). In the cardiovascular system, NOX2 and NOX4 exhibit different physiological functions such as regulating blood pressure, mediating growth factor signaling, and controlling cell proliferation. Camargo et al. (2018) demonstrated that VSMCs stimulated with AngII or VSMCs from hypertensive rats exhibit high levels of O_2_^–^ and H_2_O_2_, associated with upregulation of NOX1 and NOX4 (Camargo et al., 2018). Low NOX4 expression correlated with the inhibition of cell proliferation and the induction of senescence, but still further studies are needed to reveal the exact molecular mechanism that drives cellular senescence upon NOX4 depletion in proliferating VSMCs. Likewise, increased NOX4 expression/activity correlated with increased mitochondrial oxidative stress, mitochondrial dysfunction, and atherosclerosis in aged ApoE-KO/p47phox-KO mice (Vendrov et al., 2015). With our results, we speculate that downregulation of AngII-induced NOX expression produced by AEPs or AETa could be due to the antioxidant effect of the extracts, since it has been demonstrated that ROS-induced activation of transcription factors stimulates the production of ROS-producing enzymes (Zinkevich and Gutterman, 2011); therefore, extracts acting by reducing ROS generation could indirectly induce downregulation of AngII-trigged NOX2 and NOX4 expression.

The prevention of vascular oxidative stress and endothelial dysfunction represents reasonable therapeutic strategies in addition to the treatment of established risk factors (hypercholesterolemia, hypertension, and diabetes). There is an increase in the level of free radical peroxidation products and decreasing activity of antioxidant enzymes in tissues from animals with experimental atherosclerosis (Lankin and Tikhaze, 2017). In this study, hypercholesterolemic mice were chronically fed with AEPs or AETa during 2 months, and remarkably, both extracts lower triglyceride levels as compared with control animals, and particularly AEPs also produced an antiglycemic effect. These findings highlighted an important capacity for metabolic regulation never described before for these native plants. There are, however, other specific species of the *Prosopis* genus that have shown hypoglycemic effects and lipid-lowering properties (Sharifi-Rad et al., 2019), such as *Prosopis farcta* (Dashtban et al., 2016; Feyzmand et al., 2018), *Prosopis glandulosa* (George et al., 2011), *Prosopis nigra* (Perez et al., 2018), and *Prosopis cineraria* (Sharma et al., 2010), but to our knowledge, these effects have not been described for *P. strombulifera*.

The composition of *P. strombulifera* and *T. absinthioides* has been recently published showing a fingerprint composed of phenolic compounds, procyanidins and flavonoids (Gomez et al., 2019; Persia et al., 2020), so it was not surprising to find that the extracts of these plants were able to increase plasmatic TAS and GPx activity in the liver and to diminish plasmatic lipid peroxidation, suggesting a potent antioxidant capacity for both extracts. Surprisingly, we found an almost complete suppression of superoxide anion generation in the liver and in aortic tissue where a reduction in 8 OH-dG, a marker of oxidative damage in DNA, was also detected. These results led us to hypothesize that lessening oxidative stress in aortic tissues could cause a reduction in the development of atheroma plaques. Our data revealed that aqueous extracts of *P. strombulifera* and *T. absinthioides* produce athero-protective effects, protect the vascular wall from damage induced by oxidative stress, and prevent atheroma progression independently of elevated plasma TC. No other species of *Prosopis* or *Tessaria* have demonstrated anti-atherogenic effects such as those described for the extracts used in our study. *P. glandulosa*, for example, is cardioprotective as well as anti-hypertensive in an animal model of pre-diabetes (Huisamen et al., 2013), and *P. farcta* root produces important lipid-lowering effects in rabbits with high cholesterol diet, but microscopic studies showed that *P. farcta* treatment did not decrease the size of atherosclerotic plaques after being formed in rabbit aortic wall (Saidi et al., 2016).

A major limitation of the use of aqueous extracts from native plants could be attributed to the feasibility of using chemically standardized aqueous extracts, as we use in our experiments, in sufficient quantity for large-scale clinical trials, which is not justified by the high costs. However, the evidence showed in this work makes the aqueous extract an important material to perform bio-guided fractionation procedures destined to isolate and identify specific chemical molecules with vascular-protective relevance.

In summary, we demonstrated that AEPs and AETa are active inhibitors of VSMC proliferation by arresting the cell cycle progression on G2/M phase, and particularly, AETa triggers VSMCs to apoptosis. Both extracts are capable of increasing the total antioxidant capacity, reducing lipid peroxidation, and preventing vascular ROS generation by modulating NOX system. The combination of antimitogenic effect and antioxidant capacity of the extracts is effective to reduce atheroma development. Further studies are needed to fully understand the molecular mechanism of action of *P. strombulifera* and *T. absinthioides*, but we consider our findings are a beginning in elucidating the potential effects of these native plants.

## Data Availability Statement

The raw data supporting the conclusions of this article will be made available by the authors, without undue reservation.

## Ethics Statement

The animal study was reviewed and approved by the Animal Experimentation Committee of Facultad de Ciencias Médicas, Universidad Nacional de Cuyo. CICUAL Aval approved # 77/2016.

## Author Contributions

IQ, MP, and CC conceived and planned the experiments. IQ, MP, and MH carried out the experiments. CG-L and MH contributed to reagents and analytic tools, analyzed, and discussed the data. MA and CC drafted, wrote, and edited the manuscript. All authors reviewed critically the manuscript and had final approval of the submitted version.

## Conflict of Interest

The authors declare that the research was conducted in the absence of any commercial or financial relationships that could be construed as a potential conflict of interest.
