# Supplementary material for: Antioxidant and Anti-atherogenic Properties of Prosopis strombulifera and Tessaria absinthioides Aqueous Extracts: Modulation of NADPH Oxidase-Derived Reactive Oxygen Species
Source: Front Physiol. 2021 Jul 16;12:662833. doi: 10.3389/fphys.2021.662833 (PMC8322988; doi:10.3389/fphys.2021.662833)
Supplement: Supplementary file 1 [file Data_Sheet_1.pdf]

Supplementary Material:

2.-Material and Methods

2.1 Isolation of Vascular Smooth Muscle Cells

Aortas were digested with 2 mg /L collagenase, 2 mg/mL bovine serum albumin, and 0.35 mg /mL soybean trypsin inhibitor in Ham's F-12 medium. VSMC were cultured in DMEM/F12 (Gibco, USA 12400-016) media supplemented with 10% fetal calf serum (FCS, Biologicals Industries, USA), antibiotic-antimycotic (Gibco, USA 15240-062) and incubated at 37ºC in a humidified atmosphere of 5% CO_2. Early passage cells were used for experiments. VSMC at 80-90% confluence were made quiescent by serum deprivation (0.1% FCS-DMEN/F12) 24 h prior to experimentation.

2.2 Cell viability and proliferation

1×10^4 VSMCs were plated in 24-well plates and serum-starved for 24 h. Then, cells were treated with 2.5, 5, 10, 20 or 40 (µg/mL) of AEPS or AETA in either 0.1% or 10% FCS DMEM/F12 during 24-48 h. MTT (3-(4,5-Dimethylthiazol-2-yl)-2,5-Diphenyltetrazolium Bromide) solution (5 mM) was added 4 h before the end point and cells were incubated at 37 °C in the dark. Absorbance was measured at 570 nm in Multiskan FC (Thermo Scientific) microplate reader. A 5-point calibration curve (number of cells vs. absorbance $_{570\ nm}$) was performed to express the results as cell number per well.

2.3Measurement of Reactive Oxygen Species (ROS) in intact cells

Intracellular ROS levels were measured using the fluoroprobe CM-H_2DCFDA (Invitrogen, C400). VSMC were cultured in a 24-well plate until confluence and then serum deprived for 24 h. AEPs and AETa (2.5, 5, 10, 20 or 40 µg/mL) were applied during 1 hour. Cells were loaded with CM-H_2DCFDA 6 µmol/L, dissolved in DMSO, and incubated 30 min at room temperature. Basal fluorescene was measured and then 100 nM angiotensin II (Ang II)

(Sigma-Aldrich A9525) was added. Fluorescence of stimulated cells was measured continuously during 40 min on a microplate fluorometer (Fluoroskan Ascent, Labsystems), λ_{ex}= 485 nm and λ_{em}= 538 nm.

To determine intracellular O·−2 production in VSMCs, we used dihydroethidium (DHE; Invitrogen). In this assay, DHE is oxidized by O·−2 to ethidium and oxyethidium, producing red fluorescence indicating the relative levels of $O_2\bullet^-$ production. In brief, VSMCs were pre-incubated 1h with AEPs or AETa (5 or 20 µg/mL) and stimulated with AngII (100 nM, 2 h). Then, cells were incubated with DHE (5 µM) for 30 min at room temperature and imaged by fluorescence microscopy (Eclipse TE300 inverted microscopy, Nikon), with exposure intensity adjusted relative to control VSMCs (unstimulated). To confirm that cell incubation with extracts affect ROS generation, control experiments with antioxidant agents including apocynin (a ROS scavenger; 100 µmol/L) and diphenyl iodonium (DPI; a flavoprotein inhibitor) 10 µmol/L, were carried out.

2.4 Quantitative reverse transcription-polymerase chain reaction (RT-PCR) analysis

Total RNA was isolated with Trizol (Invitrogen, 15596026) from VSMC stimulated with AngII and treated with or without AEPs or AETa (2.5, 5, 10, 20 µg/mL). Total RNA was reversed transcribed, using random primer hexamers (Biodynamics, Argentina. B070-40), and M-MLV reverse transcriptase (Promega, USA. M1701). Real-time qPCR was performed with cDNA samples and EVA Green (Biotium, USA. 31000) using a Rotor-Gene 6000 Series Software version 1.7 (Corbett). All samples were amplified in triplicate. Oligonucleotide primers (OligoLTA, Thermofischer Scientific) were as follows: NOX2 sense: 5′-CTGTGATAAGCAGGAGTTCCAA -3′, and antisense: 5′-CCTGCACAGCCAGTAGAAGT -3′; NOX4 sense: 5′-GCTTGTTGAAGTATCAAACCAAT -3′, and antisense: 5′-TCCAGAAATCCAAATCCAGGT-3′; 18S sense: 5′-GGCTCATTAAATCAGTTATGGTTCCT-3′, and antisense 5′-

GTTGGTTTTGATCTGATAAATGCACG-3'. Optimized amplification conditions were 0.2 µM of each primer, 2.5 mM $MgCl_2$, annealing at 60°C and extension at 72°C for 40 cycles. The gene expression levels of Nox1, Nox2 and Nox4 were normalized to 18S. The mRNA levels were expressed as a ratio, using the delta-delta method for comparing relative expression results between treatments. Relative expression was calculated as $2^{-\Delta\Delta Ct}$.

2.5 Animals and study design

C57/BL6J ApoE-KO mice two months old (The Jackson Laboratories, Bar Harbor, ME) were used for this study. Mice were grouped-housed, had unrestricted access to water and standard chow (GEPSA, Argentina) and were maintained on a 12 h light/dark cycle. Mice were fed a normal diet and treated 8 weeks with AEPs or AETa (150 or 300 mg/kg/day, respectively) in the drinking water. During this time body weight, diet and water intake control were monitored. Just before the animals were euthanatized, blood samples were collected after 4 hours fasting period, by cardiac puncture under anesthesia (ketamine: 80 mg/kg-midazolan: 5 mg/kg). The glucose concentrations were measured immediately after blood collection with an enzymatic kit (Wiener Lab® Rosario, Argentina. 1400101). Plasma Total Cholesterol (TC) and Triglycerides (TG) were determined using colorimetric reactions with commercial kits (GT Lab, Buenos Aires, Argentina. 750220 and 791020 respectively). As an indicator of lipid peroxidation, plasma malondialdehyde (MDA) was determined using the TBARS assay (TBARS) in an established laboratory protocol. To this end, 0.219 ml of TBARS reagent (15% trichloroacetic acid, 0.25 N *Hydrochloric* acid, 0.6% thiobarbituric acid, Sodium Dodecyl Sulfate 8%, butylated hydroxytoluene 6%) was added to a 0.1 mL aliquot of serum (or standard curve point) and heated for 30 minutes at 95°C. After cooling the samples, 0.6 ml of butanol was added and mixed gently during 5 min and then centrifuged at 3000 rpm for 15 min. 150 µL of the supernatant from each tube was transferred onto 96 well round

bottom plates and absorbance was read at 532 nm on a Multiskan FC (Thermo Scientific) microplate reader. Blanks were prepared and analyzed in the same way to exclude any A532 contribution due to the background. The TBARS results were expressed as MDA equivalents using 1,1,3,3-tetraethoxypropane (Sigma-Aldrich **T9889)**. A standard curve was made to represent 0,1,2, 3,4,5,7.5,10 and 15 µM malondialdehyde using 100 µM 1, 1, 3, 3-tetramethoxypropane standard.

2.6 Determination of tissue Glutathione Peroxidase Activity and NOX-derived superoxide

For the antioxidant enzyme determination, liver was homogenized in a proportion of 200 mg/ mL of homogenization buffer (phosphate buffer plus Complete protein inhibitor 1000X, Roche). Subsequently, the homogenate was centrifuged at 10,000 x g for 15 minutes at 4ºC, to eliminate cellular organelle debris, and the supernatant was used for the determination of the antioxidant enzyme glutathione peroxidase (GSH-Px) activity .The protein concentration was determined following the method described by Bradford, using the Bio-Rad Protein Assay.

GSH-Px activity was determined by using a Randox laboratories commercial kit. This method is based on the ability of this enzyme to catalyze the oxidation of glutathione (GSH) by cumene hydroperoxide in the presence of glutathione reductase (GR) and NADPH. The oxidized glutathione (GSSG) returns to its reduced form at the expense of the oxidation of NAPDH; its disappearance is assessed by measuring the decrease in absorbance at 340nm. Enzyme activity was expressed as U/mg of protein.

Isolated liver and aortas were homogenized in buffer containing 8 mM potassium, sodium phosphate buffer, pH 7.0, 131 mM NaCl, 340 mM sucrose, 5 mM MgCl2, 1 mM EGTA, and protease inhibitors (Roche); and centrifuged at 10,000 rpm for 15 min at 4 °C to remove unbroken cells and debris. The assay of the supernatant was carried out using acetylated

cytochrome *C* (0.2 mM, Sigma–Aldrich C7752), in buffer containing catalase (300U/ml) to prevent re-oxidation of reduced cytochrome *C* by H_2O_2. An identical set of samples was incubated in the presence of SOD (150U/ml) for subtraction of the SOD-inhibitable signal. After 5 min baseline measurement, NADPH (180 µM) was added and $O_2^{\bullet-}$ production was measured at 550 nm using an spectrophotometer (Mutiskan FC, Thermo Fisher). Superoxide production is expressed as nmoles $O_2^{\bullet-}$ min/mg protein using the extinction coefficient.

2.7 Atherosclerotic lesions measurement

C57/BL6J ApoE-KO mice four months old were randomly divided into four groups (n = 4–6 mice each) and fed a control diet (Control), high fat diet (HFD) (standard chow supplemented with 30% bovine fat) during 8 weeks or HFD supplemented either with AEPs or AETa (150 or 300 mg/kg/day, respectively) in drinking water the last 4 weeks. All mice were sacrificed and the whole aortas were excised. Each artery was fixed with 4% paraformaldehyde for 2 h, and then placed in a 30% sucrose solution for 24 h. Following fixation, the arteries were stained with an oil red-O solution (Sigma-Aldrich, O0625) (5 mM oil red, 0.3 M NaOH, 70% MeOH) for 2 h. Then, the arteries were washed with 70 % MeOH and distilled water, and finally, were photographed using a 519CU CMOS 5.0 Megapixel Camera and Micrometrics SE Premium Software. Plaque area in the images was quantified using the Image J 1.50i software.

2.8 Immunofluorescence staining

Sections (5 µM) of 4%paraformalin-fixed, paraffin-embedded aortic tissues were subjected to immunofluorescence staining. Autofluorescence was eliminated by exposure to UV light for 30 minutes. The sections were incubated with 0.3% Triton X-100 (Sigma-Aldrich) for 5 min at room temperature and washed three times with PBS. The sections were then blocked with 1% calf serum for 1 h at room temperature and incubated primary antibodies against 8-

OHdG conjugated to fluorescein isothiocyanate (FITC) (Abcam ab183393 1:500) overnight at 4°C. The stained specimens were mounted and observed under confocal fluorescence microscope (Olympus FV-1000).

Table 3

The body weights of mice in each group before experiment and after feeding for AEPs or AETa during 8 weeks (g)

Group	n	Before	After
Control chow	9	20.00 ± 1.07	21.25 ± 1.16
AEPs	9	19.75± 1.21	21.13 ± 0.99
AETa	9	21.0 ± 1.89	21.75 ± 1.75

The body weights before experiment and after feeding HFD for 12 weeks and AEPs or AETa for 4 weeks (g)

Group	n	Before	After
Control chow	6	29.33 ± 0.33	31.20 ± 0.33*
HFD	6	29 ± 0.82	35.80 ± 1.54**
HFD + AEPs	6	28.67 ± 1.15	34 ± 1.18**
HFD +AETa	6	28.5 ± 0.56	32.20 ± 0.62**

*P < 0.05, **P<0.01 vs initial weight (before)
